# Myotonic dystrophy type 1 patient-derived iPSCs for the investigation of CTG repeat instability

**DOI:** 10.1038/srep42522

**Published:** 2017-02-13

**Authors:** Junko Ueki, Masayuki Nakamori, Masahiro Nakamura, Misato Nishikawa, Yoshinori Yoshida, Azusa Tanaka, Asuka Morizane, Masayoshi Kamon, Toshiyuki Araki, Masanori P. Takahashi, Akira Watanabe, Nobuya Inagaki, Hidetoshi Sakurai

**Affiliations:** 1 Center for iPS Cell Research and Application (CiRA), Kyoto University, 53 Shogoin-Kawahara-cho, Sakyo-ku, Kyoto 606-8507, Japan; 2 Department of Diabetes, Endocrinology and Nutrition, Graduate School of Medicine, Kyoto University, 54 Shogoin-Kawahara-cho, Sakyo-ku, Kyoto 606-8507, Japan; 3 Department of Neurology, Osaka University Graduate School of Medicine, 2-2 Yamadaoka, Suita, Osaka 565-0871, Japan; 4 Department of Peripheral Nervous System Research, National Institute of Neuroscience, National Center of Neurology and Psychiatry, 4-1-1 Ogawa-Higashi, Kodaira, Tokyo 187-8502, Japan; 5 Department of Functional Diagnostic Science, Osaka University Graduate School of Medicine, 1-7 Yamadaoka, Suita, Osaka 565-0871, Japan

## Abstract

Myotonic dystrophy type 1 (DM1) is an autosomal-dominant multi-system disease caused by expanded CTG repeats in dystrophia myotonica protein kinase (*DMPK*). The expanded CTG repeats are unstable and can increase the length of the gene with age, which worsens the symptoms. In order to establish a human stem cell system suitable for the investigation of repeat instability, DM1 patient-derived iPSCs were generated and differentiated into three cell types commonly affected in DM1, namely cardiomyocytes, neurons and myocytes. Then we precisely analysed the CTG repeat lengths in these cells. Our DM1-iPSCs showed a gradual lengthening of CTG repeats with unchanged repeat distribution in all cell lines depending on the passage numbers of undifferentiated cells. However, the average CTG repeat length did not change significantly after differentiation into different somatic cell types. We also evaluated the chromatin accessibility in DM1-iPSCs using ATAC-seq. The chromatin status in DM1 cardiomyocytes was closed at the *DMPK* locus as well as at *SIX5* and its promoter region, whereas it was open in control, suggesting that the epigenetic modifications may be related to the CTG repeat expansion in DM1. These findings may help clarify the role of repeat instability in the CTG repeat expansion in DM1.

Myotonic dystrophy type 1 (DM1) is a chronic, slowly progressive, autosomal-dominant and multisystem disease^1^. DM1 is caused by expanded CTG repeats in dystrophia myotonica-protein kinase (*DMPK*). Its symptoms, such as myotonia, muscle wasting and cardiac conduction defects, have been thought to be the result of splicing defects caused by toxic mRNA that includes expanded CUG repeats^2^. The expanded CTG repeats in DM1 patients are unstable and can reach several thousand CTG repeats. In addition, the continuous growth of the expanded repeats can affect the progression of the symptoms^3^. Furthermore, the repeat size can vary between tissues (somatic instability), and the main affected tissues in DM1, such as neurons as well as skeletal and cardiac muscles, normally show longer CTG repeats than other tissues^4^,^5^,^6^,^7^.

Previous studies have confirmed these phenotypes in patient-derived tissues and cell models, but have yet to explain the mechanism causing the expansion of the CTG repeats or the reason why the expansion is more apparent in specific tissues. This may be due to the difficulty in acquiring a sufficient number of DM1 patient cells, especially from neural or cardiac tissues.

However, this problem can be remedied by using patient-derived induced pluripotent stem cells (iPSCs), which have made it possible to study several diseases that refer to the cell types commonly targeted by DM1^8^,^9^,^10^,^11^. Additionally, protocols for differentiation into cardiomyocytes (CMs), neurons and myocytes from iPSCs with high efficiency and stability have also been established^10^,^11^,^12^,^13^.

There is only one previous study on DM1 using iPSCs, which showed that the peak length of the CTG repeats increased with the passaging of the cells, but not after their differentiation into neurospheres^14^. Although the results recapitulate the gradual increase of repeat length during propagation, as seen in DM1 fibroblasts^15^, the changes in repeat-size distribution during propagation or after differentiation were not clear. Another study using human embryonic stem cells (hESCs) showed that the peak length of the CTG repeats and repeat size distribution changed with passaging, but that it stabilized once the cells were differentiated into osteoprogenitor-like cells^16^. However, in the previous study, the repeat size changes varied among the clones, and the tendencies toward contraction or expansion were not consistent. Therefore, in this study, to establish a human stem cell system suitable for the investigation of repeat instability, we acquired iPSCs from DM1 patients. We then analysed repeat instability not only in the undifferentiated state but also in the differentiated cells which form the main affected tissues of DM1. We then analysed repeat instability by means of small-pool PCR (spPCR) not only in the undifferentiated state but also in the differentiated cells which form the main affected tissues of DM1. SpPCR is so far the best way to evaluate all the different CTG repeat lengths in a tissue with heterogeneity^17^. In spPCR, the DNA is diluted to the equivalent of a small number of genomes before amplification. This dilution allows the identification of PCR products derived from single-input molecules by agarose gel electrophoresis and Southern blot hybridization. This technique provides detailed information about the repeat-length variation in a sample, including rare large contracted or expanded alleles as well as the repeat size distribution, whereas conventional bulk PCR just gives the peak length of all the different repeats.

Furthermore, several lines of evidence suggest that the epigenetic modifications may be related to the CTG/CAG repeat instability in DM1^18^. Epigenetic regulations, such as DNA methylation and chromatin structure, play a central role in gene expression. The open chromatin regions indicate the transcriptionally active regions. We next address the epigenetic regulation, which affects the gene expression profile. We performed Assay for Transposase-Accessible Chromatin using sequencing (ATAC-seq), which detects the transcriptionally active open chromatin regions. ATAC-seq requires very low numbers of cells (~1000 s cells)^19^. The number of cells of our iPSC-CMs was very limited. However, ATAC-seq could be successfully performed with such a low number of cells. With the result, we obtained the differential open chromatin status at the gene regulatory region of *SIX5* between DM1-CMs and the control CMs.

## Results

### Generation of DM1-iPSCs and their differentiation into functional CMs, neurons and myocytes

**Figure 1:**
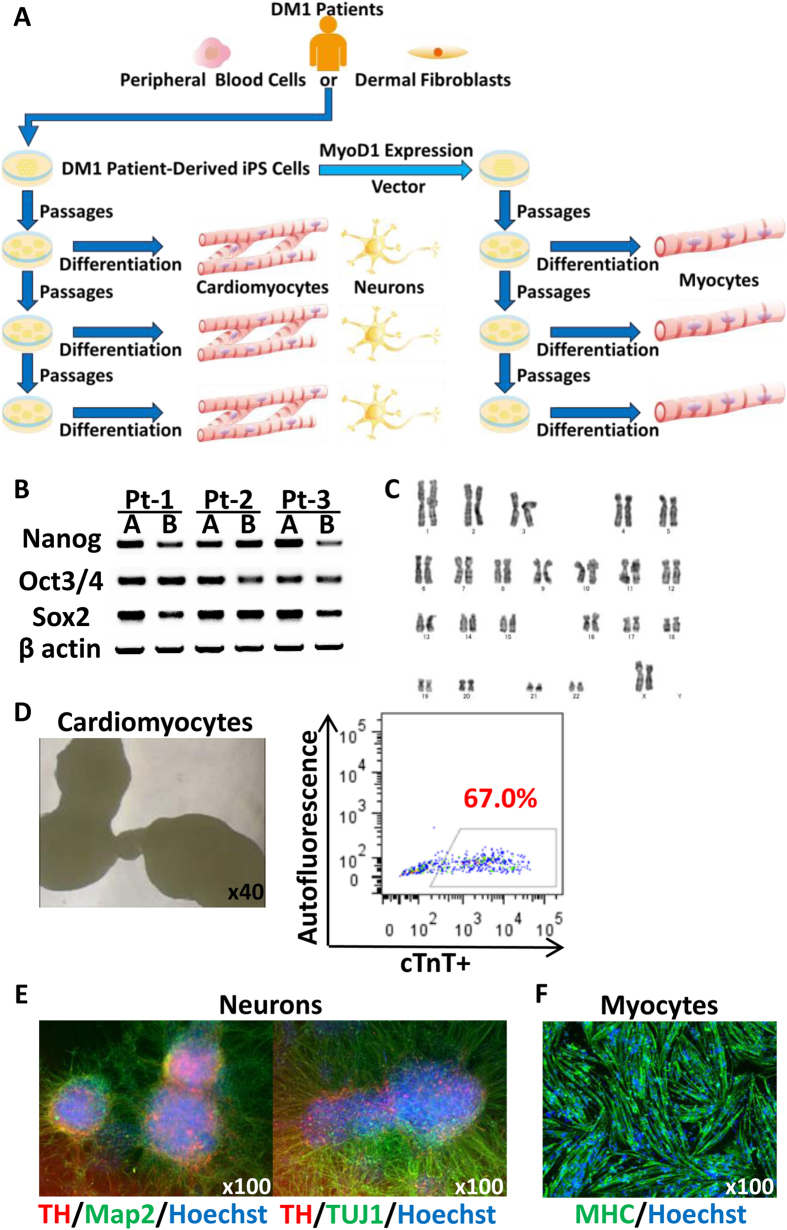
Generation of DM1-iPSCs and their differentiation. (**A**) The strategy of our study: patient iPSCs were passed and differentiated at three different passage numbers into CMs or neurons giving 9 samples (left), or had a MyoD1 vector transfected and were differentiated into myocytes, giving 6 samples (right). The CTG repeat lengths were measured in each sample. (**B**) Six clones from three different DM1 patients expressed pluripotent stem cell markers (Oct3/4, Nanog and Sox2) in conventional PCR. β-actin was used as a loading control. (**C**) Karyotypic analysis of undifferentiated iPSCS (Pt-1B). (**D**, **left**) Representative live image of CMs on day 20 (Pt-1B). A video clip is available in Supplementary Video 1. (**D**, **right**) FACS analysis of the CMs shown in the picture on the left. The X-axis indicates the percentage of cardiac troponin T (cTnT)-positive cells among the total number of CMs. The Y-axis indicates the autofluorescence of the CMs. (**E**) Representative immunostaining image of neurons on day 42 (Pt-1B). The left panel shows neurons that expressed Tyrosine Hydroxylase (TH) and Microtubule-associated protein 2 (Map2). The right panel shows neurons that expressed TH and Neuron-specific Class III β-tubulin (TUJ1). (**F**) Representative immunostaining image of myocytes on day 7 (Pt-1B). The myocytes expressed Myosin Heavy Chain (MHC). Hoechst stains the nuclei.

The six iPS clones from the three different DM1 patients expressed the pluripotent stem cell markers Oct3/4, Nanog and Sox2 (Fig. 1B), but did not show any episomal integration (data not shown) or karyotypic abnormalities (Fig. 1C).

CMs differentiated from Pt-1B showed embryoid bodies (EBs) (Fig. 1D) and a heartbeat (Supplementary Video 1). The ratio of cardiac troponin T (cTnT)-positive cells to the total number of CMs was 67% according to FACS (Fig. 1D), and for all clones it ranged between 56.1% and 89.4% (*n* = 10), which was averaged 72.7 ± 11.2% (data not shown). Immunostaining showed that neurons differentiated from Pt-1B expressed Tyrosine Hydroxylase (TH), Neuron-specific Class III β-tubulin (TUJ1) and Microtubule-associated protein 2 (Map2) (Fig. 1E), indicating that they were dopaminergic neurons. Immunostaining of neurons from the other five clones showed similar results (data not shown). More than 90% of myocytes expressed Myosin Heavy Chain (MHC) (Fig. 1F shows a result for Pt-1B).

**Figure 2:**
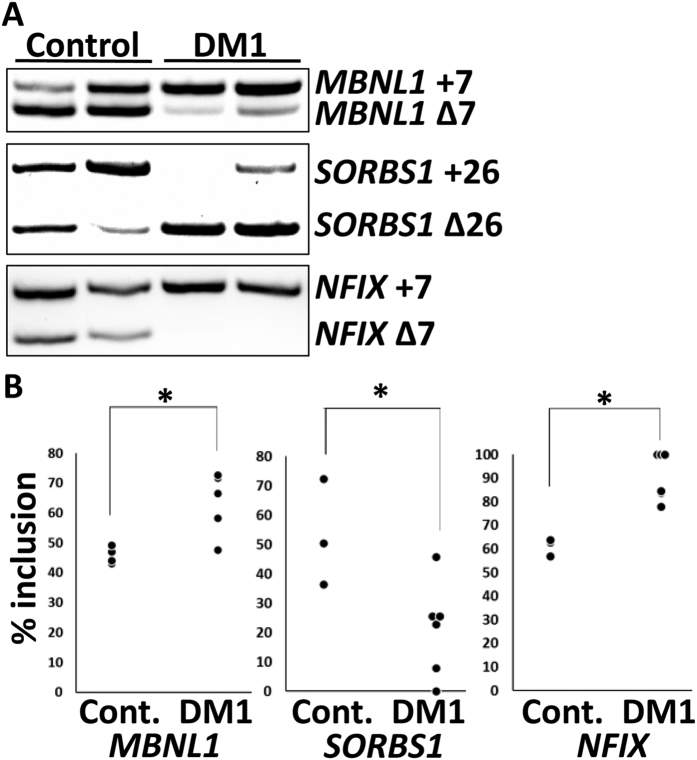
Splicing defects in differentiated cells from control iPSCs and DM1-iPSCs. (**A**) Representative reverse transcription polymerase chain reaction (RT-PCR) results of control CMs (Control, *n* = 2) and DM1-CMs (DM1, *n* = 2). The measured exons are indicated on the right. (**B**) Exon inclusion values. The Y-axis indicates the percentage of exon-including bands versus the number of exon-including and -skipping bands. Cont. = control cells. Student’s *t*-test was used to compare the control and DM1 groups (*P < 0.05). *MBNL1*: Cont. CMs, *n* = 4, DM1 CMs, *n* = 6; 10 RT-PCR products were loaded on the gel at the same time. *SORBS1*: Cont. neurons, *n* = 3, DM1 neurons, *n* = 6; 9 RT-PCR products were loaded on the gel at the same time. *NFIX*: Cont. myocytes, *n* = 3, DM1 myocytes, *n* = 6; 9 RT-PCR products were loaded on the gel at the same time.

Splicing defects in the three differentiated cell types, namely *MBNL1* exon 7 in CMs, *SORBS1* exon 26 in neurons and *NFIX* exon 7 in myocytes, were observed (Fig. 2). The three or four controls were compared with the six DM1-iPS clones to identify the cell phenotypes of DM1. In each cell type, the control samples showed a splicing pattern close to that seen in normal adult samples, while the DM1 samples resembled the patterns of DM1 (Fig. 2A)^20^,^21^. The DM1 group also showed a statistically significant difference from the control group regarding % exon inclusion (Fig. 2B). These results indicated that the differentiation from DM1-iPSCs was successful and that the splicing defects observed showed cell phenotypes consistent with DM1.

### Repeat instability

**Figure 3:**
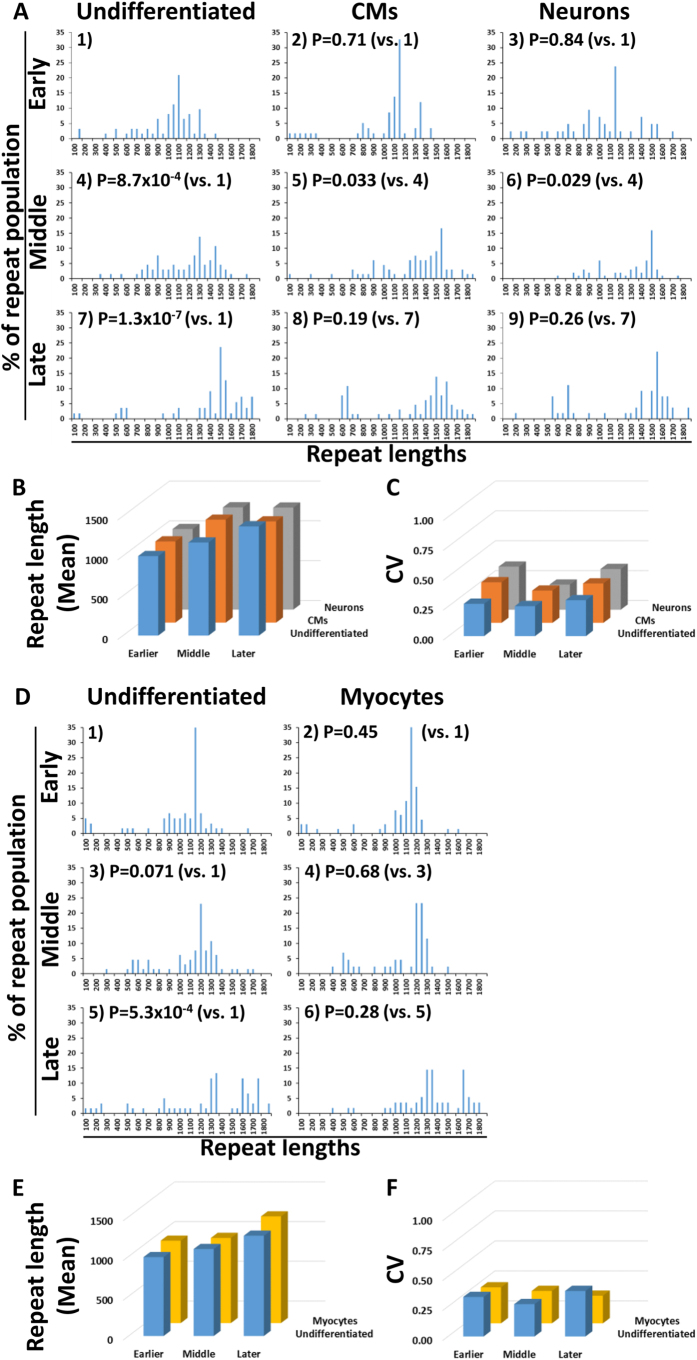
CTG repeats of Pt-1B. (**A**) The distribution of the CTG repeats in the undifferentiated iPSCs and the CMs and neurons differentiated from the undifferentiated iPSCs at early (passages 10–17), middle (passages 21–27) and late passage numbers (passages 31–37), following the strategy shown in Fig. 1A, left. The lengths of the CTG repeats were grouped in bins spanning 50 repeats. Student’s *t*-test was applied to each group of different CTG repeat lengths before being grouped in bins. *P*-values are shown. Because of the multiple comparison, the appropriate significance level was determined by Bonferroni correction, requiring a P ≤ 0.0056 to be significant at the 95% level. P stands for *P*-values. (**B**) Mean repeat length of the nine samples. The original lengths before being grouped in bins were used to calculate the mean. The nine bar graphs correspond to 1) to 9) in (**A**). (**C**) Coefficient of variation (CV) of the repeat lengths of the nine samples. CV is defined as the ratio of the standard deviation (SD) to the mean. The original lengths before being grouped in bins were used to calculate the SD. The nine bar graphs correspond to 1) to 9) in (**A**). (**D**) The distribution of the CTG repeats in undifferentiated MyoD-iPSCs and in myocytes differentiated from the undifferentiated MyoD-iPSCs at early (passages 20–26), middle (passages 29–36) and late passage numbers (passages 40–48), following the strategy shown in Fig. 1A, right. The lengths of the CTG repeats were grouped in bins spanning 50 repeats. Student’s *t*-test was applied to each group of different CTG repeat lengths before being grouped in bins. *P*-values are shown. Because of the multiple comparison, the appropriate significance level was determined by Bonferroni correction, requiring a P ≤ 0.0083 to be significant at the 95% level. P stands for *P*-values. (**E**,**F**) Mean and CV repeat lengths of six samples. The six bar graphs correspond to 1) to 6) in (**D**).

The monocytes of patient 1 showed different lengths of CTG repeats, ranging from 200 to 1,950 (Supplementary Fig. S2). Using spPCR to analyse the CTG repeat lengths precisely, one iPS clone (Pt-1B) showed a distribution of the different lengths ranging from 150 to 1,500, and a peak length of about 1,000 repeats at the passage number 10 (Fig. 3A, undifferentiated 1). The range of the lengths at the early passage numbers of the undifferentiated iPSCs and of their sources in three other clones can be seen in Table 1.

Following the strategy shown in Fig. 1A, we first analysed the CTG repeats of Pt-1B. In order to demonstrate the change of the CTG repeat lengths with the passaging of the undifferentiated iPSCs, we compared the CTG repeat lengths in undifferentiated iPSCs at the early passage number with those at the middle and late passage numbers. The average length of the CTG repeats increased significantly with passaging (Fig. 3A,B and Table 2), but the variation of the lengths was unchanged (Fig. 3C and Table 2). Also, in order to demonstrate the change of the CTG repeat lengths after differentiation from iPSCs, we compared the CTG repeat lengths in CMs with those in the undifferentiated iPSCs, from which the CMs were differentiated, at each passage number. No statistically significant difference in the average length or the variation of the lengths was found between the CMs and the undifferentiated iPSCs (Fig. 3A–C and Table 2). The comparison between the neurons and the undifferentiated iPSCs, from which the neurons were differentiated, was consistent with the comparison between the CMs and the undifferentiated iPSCs (Fig. 3A–C and Table 2). Incidentally, a smaller, second peak length appeared after the differentiation into CMs and neurons at the late passage number (Fig. 3A, bottom).

Regarding the differentiation into myocytes, the average length of the undifferentiated MyoD-iPSCs increased significantly with passaging (Fig. 3D,E and Table 3), but the variation remained unchanged (Fig. 3D,F and Table 3), which was consistent with the results of the undifferentiated iPSCs. The same was also true in the comparison between the myocytes and the undifferentiated MyoD-iPSCs, from which the myocytes were differentiated (Fig. 3D–F and Table 3).

Similar results were found for Pt-1A, which was derived from the same patient as Pt-1B (Supplementary Fig. S3, Supplementary Table S1). The other four clones, which were derived from the other two patients, were investigated using the same protocol. However, the results of Pt-2A and Pt-3A were omitted because the dominant lengths of their CTG repeats were beyond the range of accurate evaluation by spPCR. Two clones that we could evaluate by spPCR, Pt-2B and Pt-3B, showed the same tendency as the clones from patient 1. All the results of Pt-1A, Pt-2B and Pt-3B except some MyoD-iPSCs pointed to the same conclusion as the results of Pt-1B (Supplementary Figs S3–S5, Supplementary Table S1). In some of the undifferentiated MyoD-iPSCs, the average length of the CTG repeats did not increase with passaging (Supplementary Figs S3–S5, Supplementary Table S2). This might be due to the random integration of the piggyBac vectors in the process of generating doxycycline-inducible iPSCs, as the integration might have occurred in genes which relate to cell propagation.

In order to assess the relationship between the CTG repeat expansion and the number of cell divisions, we performed the cell division assay (Supplementary Fig. S6A–C). The number of cell divisions of Pt-1B-MyoD undifferentiated iPSCs after culturing for seven days was averaged 8.9 ± 0.2 times (*n* = 3) and that of Pt-1B-MyoD myocytes seven days after differentiation was averaged 5.7 ± 0.9 times (*n* = 3) (Supplementary Fig. S6D). The doubling time of Pt-1B-MyoD undifferentiated iPSCs in the undifferentiated culture was averaged 19 ± 0.48 hours (*n* = 3) and that of Pt-1B-MyoD myocytes in the myogenic differentiation culture was averaged 30 ± 4.5 hours (*n* = 3) (Supplementary Fig. S6E). We confirmed that the number of cell divisions was higher in the undifferentiated iPS cell culture than in the myogenic differentiation culture.

In short, the average length of the CTG repeats increased, but the variation of the CTG repeat lengths was unchanged with the passaging of the undifferentiated iPSCs. Neither the average length nor the variation of the CTG repeat lengths changed significantly after the differentiation from the undifferentiated iPSCs into the three differentiated cell types.

### Chromatin accessibility

**Figure 4:**
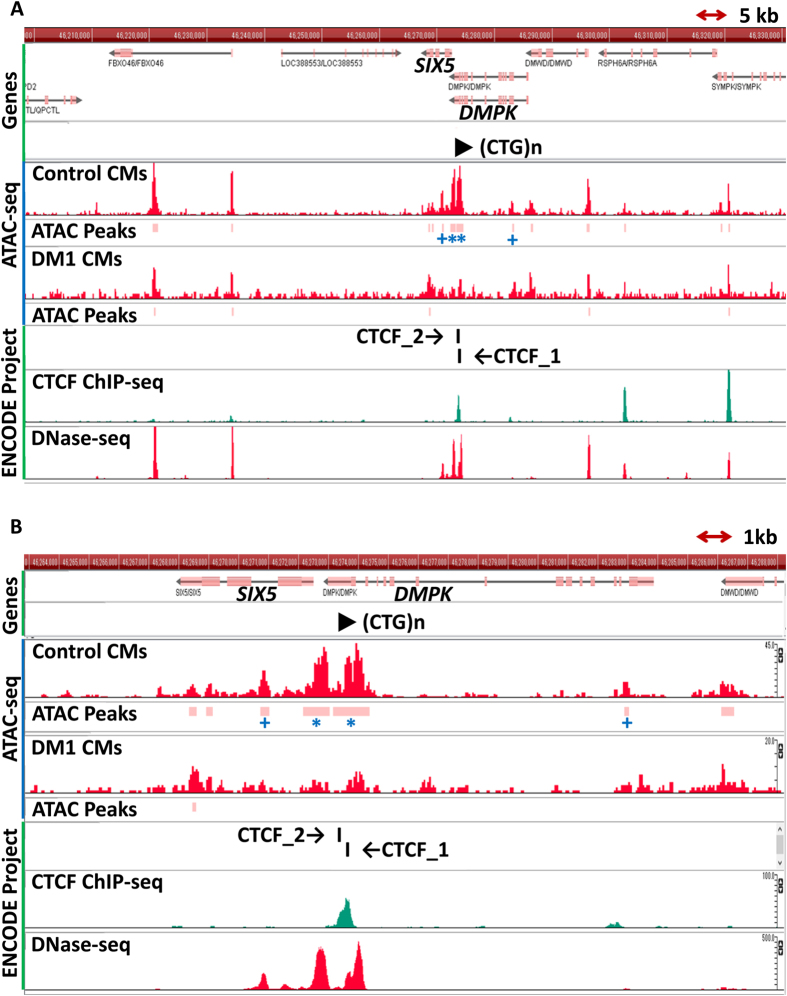
ATAC-peaks corresponding to the area around *DMPK*. (**A**) GenomeJack Browser tracks show ATAC-peaks of control CMs and DM1-CMs (red tracks, control and DM1) as well as CTCF ChIP-seq (green track) and DNase-seq (red track, bottom) of CMs from the database of ENCODE project. Pink bars indicate ATAC peaks of control CMs. ^+^ and *indicate differential ATAC peaks between control CMs and DM1-CMs (trimmed mean of M-values (M) >1: tag ratio after normalization >2; ^+^*p* < 0.01, *p < 10^−5^). (**B**) Magnified image of A in the area around *DMPK*: differential ATAC peaks shown by ** are located between CTCF site 1 and CTCF site 2.

In order to investigate the structural changes in chromatin on the expanded allele directly, ATAC-seq was conducted on the control CMs derived from one healthy iPS clone and one DM1-iPS clone (Fig. 4). For the control CMs, most of the regions in the genome and the pattern of the ATAC peaks were consistent with DNase I hypersensitive site sequencing (DNase-seq) peaks of healthy CMs in the database of the ENCODE project (Fig. 4, DNase-seq). This result confirmed that ATAC-seq could detect if the chromatin structure is open or closed, as did DNase-seq in the previous studies, and that our control CMs can function as a healthy control in the ATAC-seq data. Focusing on the regions around *DMPK* (Fig. 4B), the comparison between the CTCF Chromatin Immunoprecipitation sequencing (ChIP-seq) data in the database of the ENCODE project and our control ATAC data showed that the ATAC peaks of our control CMs are located in the regions flanking the functional CTCF binding sites in *DMPK* (Fig. 4B, CTCF_1 and CTCF_2). On the other hand, some of the ATAC peaks of DM1-CMs were lower than those of the control CMs (Fig. 4A and B, Control vs. DM1). Interestingly, a quantitative assessment using MAnorm revealed that the DM1-CMs showed significantly lower ATAC peaks in the area of *DMPK* including CTCF binding sites (Fig. 4A,B, ^+^*p* < 0.01 and *p < 10^−5^). In addition, lower ATAC peaks can be seen in *SIX5* and its promoter region (Fig. 4A,B, ^+^ and *). These results indicate that the chromatin around the expanded CTG repeats in the DM1-CMs was closed. We compared ATAC-seq peaks of the control CMs and DM1-CMs at a genome wide scale and observed that many chromatin regions were closed in DM1-CMs. The 7,500 peaks were decreased and the 486 peaks were increased in DM1-CMs compared to the control CMs.

## Discussion

CTG repeat expansion occurs throughout the life of an individual with DM1^3^. Our DM1 iPSCs showed a gradual increase in the average repeat length during propagation. The average lengthening of the CTG repeats with the passaging of the undifferentiated iPSCs could suggest that the cell propagation drives the CTG repeat lengthening. It is true that cardiac muscles, neurons and skeletal muscles are the main affected tissues in DM1 adults, and the CTG repeats in these tissues are longer than those of other adult tissues^7^. However, our results showed that the dominant CTG repeat lengths in our iPSCs differentiated into myocytes or neurons were consistent with those in the undifferentiated iPSCs. Based on the results of doubling time, we calculated that the undifferentiated iPSCs divided 50.5 times during 10-time passaging for 40 days, while the myocytes divided 8 times after the 10-day differentiation. This difference of the cell division rate might affect the higher repeat expansion of the undifferentiated iPSCs in our experiments. Conversely, our results also demonstrated that the differentiation into specific lineages does not affect the repeat expansion. However, the DM1 patients have different CTG repeat lengths in their tissues/organs. It is also possible that some DNA repair mechanisms play a role in the CTG repeat expansion in the tissues of the DM1 patients during aging^22^,^23^. However, the differentiation method of iPSCs which can induce enough mature tissues to mimic the aging and DNA repair process in the human body has not been established yet. We believe that further investigation is needed after establishing the methods of cell culture which reproduce the DNA repair. Incidentally, for Pt-1B at a later passage number, the second peak length appeared after the differentiation into CMs and neurons (Fig. 3A, bottom). This might be the case because the cells with shorter CTG repeats were more easily differentiated under some conditions.

From the early passage number, DM1-iPSCs showed different CTG repeat lengths, whose variation was a peak length and smaller number of the shorter and longer lengths. The variation of the expanded CTG repeat lengths was unchanged with passaging or after differentiation, once that variation was achieved. This was seen in all of the clones, which we generated. The consistent variation is surprising considering that our iPS clones were built using a colony derived from one cell, which was supposed to have one expanded CTG allele and one normal allele, suggesting that the expanded CTG repeats were unstable from the very beginning of the iPS clone generation. In contrast to a previous DM1 cell model showing increased variability with an unchanged average repeat length^24^, our DM1-iPS cell model presents a gradual lengthening of the expanded repeats with unchanged variability. That pattern is quite similar in myoblasts derived from DM1 patients^25^, hence, our iPS cell model would be suitable for studying the mechanism of repeat expansion during cell propagation in DM1.

A previous study reported that hESCs with the longer CTG repeats showed a decreasing expression level of *SIX5*, accompanied by an increasing DNA methylation level on the promoter^26^. In addition, DM1 patient-derived tissues showed high CpG methylation in the regions flanking the expanded repeats, including a CTCF binding site, compared with healthy cells^4^, and the histone mark of the CTCF binding regions changed in accordance with the expansion of the CTG repeats in model mice^27^. However, the chromatin status as a result of many different modifications had not been studied directly yet. ATAC-seq data showed that the chromatin status in DM1-CMs was closed at *SIX5/DMPK* locus, whereas it was open in control CMs. A genome-wide survey of the DM1-CMs-specific chromatin status also demonstrated that the number of the closed chromatin regions was higher than that of the open chromatin regions. It remains unknown how the closed chromatin status in DM1 affects the disease procession. The closed chromatin status might contribute to the disease severity because abnormal CpG methylation upstream the CTG repeat tract is often observed in the most severe congenital forms of DM1. On the other hand, the closed chromatin status could be the result of the defence mechanism to moderate the symptoms of DM1, such as a suppressed transcription of toxic RNA from the expanded CTG repeats, although the defence mechanism is not good enough to remove the symptoms. It remains unknown whether the differential chromatin status is the cause or the result of the CTG repeat expansion. A further investigation of the role of the epigenetic changes would help uncover the molecular pathology of DM1.

## Methods

### Study ethics

This study was approved by the Ethics Committees of the Graduate School of Medicine Kyoto University, the Kyoto University Hospital, the Osaka University Graduate School of Medicine and the National Center for Neurology and Psychiatry. Written informed consent was obtained from the patients in accordance with the Declaration of Helsinki. All methods were performed in accordance with the relevant guidelines and regulations approved by the Institutional Review Board at Kyoto University.

### Generation of DM1-iPSCs and their differentiation into functional CMs and neurons

DM1-iPSCs were generated from three female adult patients. Two iPS clones from the peripheral blood were obtained from one patient, and four iPS clones from the dermal fibroblasts of two other patients. All clones were generated using episomal vectors as described previously^28^,^29^. A list of the clones is shown in Table 1. The iPSCs were cultured in Primate ES Cell medium (Repro CELL, Kanagawa, Japan) supplemented with 8 ng/ml basic fibroblast growth factor (bFGF, Wako, Osaka, Japan) and 50 U/l penicillin/50 μg/l streptomycin (Nacalai Tesque, Kyoto, Japan), as described previously^30^. The DM1-iPSCs were transferred at a split ratio of 1: fewer than or equal to 4 as clusters to 60 mm dishes, mostly every 4 days, after the cells covered 70–80% of the dishes. For each clone, while culturing the undifferentiated iPSCs, the differentiation into CMs or neurons was conducted at three different passage numbers separated by about 10 passages: passages 10–17 (early), passages 21–27 (middle) and passages 31–37 (late). CMs were differentiated from the undifferentiated iPSCs after about 20 days of culturing, and neurons after about 42 days. The differentiation into CMs from the DM1-iPSCs and healthy control iPSCs (201B7, 648A1 and 606A1) was conducted as described previously^12^, as was the differentiation into neurons from the DM1-iPSCs and healthy control iPSCs (836B1, 404C2 and 1231A1)^13^.

### Generation of doxycycline-inducible MyoD-DM1 patient-derived iPSCs (MyoD-DM1-iPSCs)

MyoD1 expression vectors were transferred to each iPS clone at around passage number 10, thus making the expression of MyoD in each clone doxycycline-inducible, as described previously^10^,^11^. The MyoD-DM1-iPSCs were cultured in Primate ES Cell medium supplemented with 8 ng/ml bFGF (Wako), 100 μg/ml neomysin (Nacalai Tesque) and 50 U/l penicillin/50 μg/l streptomycin (Nacalai Tesque). The MyoD-DM1-iPSCs were transferred in the same way as the DM1-iPSCs. The bulk cultures of all initial colonies of MyoD-DM1-iPSCs were used to differentiate myocytes, although the selection of a few high mCherry-expressed clones after the transfer of the MyoD1 expression vector was needed to differentiate myocytes efficiently in previous studies. The differentiation into myocytes was conducted at three different passage numbers: passages 20–26 (early), passages 29–36 (middle) and passages 40–48 (late).

### Differentiation into myocytes

MyoD-DM1-iPSCs were passed four or five times using S-Medium (Sumitomo Dainippon Pharma, Osaka, Japan) following the manufacturer’s protocol until the SNL feeders were completely removed. Then, a 24-well plate (AGC TECHNO GLASS, Shizuoka, Japan) was coated with Matrigel (Becton, Dickinson and Company, New Jersey, USA) diluted 1:50 volume with S-Medium while incubating at 37 °C with 5% CO_2_ for 120 min, and the solution was aspirated just before the cell transfer. After following the manufacturer’s protocol of passage, the cells were suspended in S-Medium supplemented with 10 μM Y-27632 (Nacalai Tesque) without bFGF and dissociated into single cells by pipetting five times. The cells were seeded at 1.5 × 10^5^ cells per well of the 24-well plate. The incubation was kept at 37 °C with 5% CO_2_ and the cells were observed every 24 hours at the time the medium was added or changed. Initially, after 24 hours (at the beginning of day 1), the medium was changed to S-Medium supplemented with 500 ng/ml doxycycline (Dox; LKT Laboratories, Minnesota, USA) three times, then at the beginning of day 4, the medium was changed to the differentiation medium, which was alpha Minimal Essential Medium (αMEM; Nacalai Tesque) with 10% Knockout Serum Replacement (KSR; Invitrogen, California, USA), 50 u/l penicillin/50 μg/l streptomycin (Nacalai Tesque), 500 ng/ml Dox (LKT Laboratories) and 100 μM 2-Mercaptoethanol (2-ME; SIGMA-ALDRICH, Missouri, USA) three times. On day 7, genomic samples and RNA samples were collected, and the cells were fixed. This protocol worked well in only some of the clones, but not in others. Therefore, the protocol was later modified as follows. After the first 24 hours, 1 ml of S-Medium was added to the wells. From the beginning of day 2, the medium was changed to S-Medium until the cells covered about 70–90% of the well. Once this was achieved, the medium was changed to S-Medium supplemented with 500 ng/ml Dox (LKT Laboratories) once or twice. After that, the medium was changed to the differentiation medium at least three times and up to seven times. Finally, after a certain number of myocytes were observed, genomic samples and RNA samples were collected, and the cells were fixed.

### Fluorescence-activated cell sorting (FACS)

The maturation of functional CMs was ascertained using FACS analysis, as described previously^12^,^31^; however, the duration for the dissociation of CMs with collagenase I was only one hour in our study.

### Immunofluorescence staining

Neurons and myocytes were fixed using 4% paraformaldehyde (PFA)/phosphate buffered saline (PBS) for 10 minutes at 4 °C and blocked with Blocking One (Nacalai Tesque) for more than 30 minutes at 4 °C after washing the samples in PBS twice. Next, the samples were incubated with primary antibodies diluted in 5% Blocking One/0.01% Tween-20 (Santa Cruz Biotechnology, Texas, USA)/PBS (PBS-T) for 12–18 hours at 4 °C and washed twice in PBS-T. After adding the secondary antibodies and Hoechst (1:200; Life Technologies, California, USA) diluted in 5% Blocking One/PBS-T, the samples were incubated for one hour at room temperature under a light-shield, washed two to three times in PBS-T and observed with a BZ-9000E fluorescence microscope (Keyence, Osaka, Japan). The primary and secondary antibodies were as follows: Mouse Anti-Human Myosin Heavy Chain (MHC) Monoclonal Antibody (1:400, #MAB4470, R&D Systems, Minneapolis, USA), Anti-Tubulin β3 Antibody (TUJ1) (1:1000; #MMS-435P, BioLegend, California, USA), Anti-Tyrosine Hydroxylase Antibody (TH) (1:400, #AB152, Merck Millipore, Darmstadt, Germany), Monoclonal Anti-MAP2 (2a + 2b) antibody produced in mice (MAP2) (1:500, #M1406, SIGMA-ALDRICH), Goat anti-Mouse IgG (H + L) Secondary Antibody, Alexa Fluor^®^ 488 conjugate (1:500, #A-11001, Invitrogen), Goat anti-Rabbit IgG (H + L) Secondary Antibody and Alexa Fluor^®^ 568 conjugate (1:500, #A-11011, Invitrogen).

### Genomic DNA isolation and purification

Genomic DNA was extracted from each sample using Gentra Puregene Cell Kit (QIAGEN, Hilden, Germany). In the case of undifferentiated cells, after the removal of feeder cells using Dissociation Solution for human ES/iPS Cells (Repro CELL), and in the case of the neurons and myocytes, after washing the samples in PBS once, Cell Lysis Solution (QIAGEN) was added to the cells directly, and the samples were collected using a cell scraper (AGC TECHNO GLASS). In the case of CMs, after the collection of EBs with medium and the aspiration of the supernatant after centrifugation, the EBs were washed with PBS once, and the Cell Lysis Solution was added. All samples were incubated at 37 °C for three to seven days until the resolution of the cluster was completed. Then, genomic DNA was purified following the manufacturer’s protocol. Finally, the genomic samples were quantified using Qubit (Thermo Fisher Scientific, Massachusetts, USA), diluted to about 3 ng/μl in TE buffer (pH 8.0) and stored at 4 °C.

### Repeat length analyses

The lengths of the CTG repeats were measured using spPCR followed by southern blot, as described previously^24^. A LAS 4000 CCD camera was used to detect southern blot bands (Fujifilm, Tokyo, Japan). The electrophoresis condition for the samples derived from patient 1 was 1% gel, 70 V and 4 hours, and that for the samples derived from patients 2 and 3 was 0.6% gel, 30 V and 11.5 hours. More than 41 alleles were analysed for each sample group (see Fig. 1A for the groups), as described previously^32^,^33^. Representative pictures taken with LAS 4000 are shown in Supplementary Fig. S1.

### Statistics

Student’s *t*-test and Bonferroni correction were used to analyse CTG repeat instability statistically. Two-way analysis of Variance (ANOVA) was used for the cell proliferation analysis based on Scheffe’s test. The different statistical analyses were conducted for the ATAC-seq data. (see “ATAC-seq” below).

### RNA isolation and reverse transcriptase (RT)-PCR

The total mRNA was isolated using Sepazol (Nacalai Tesque) according to the manufacturer’s protocol. The collection and suspension of the cells in Sepazol was carried out according to the DNA purification method described above. Homogenization was performed using 29 gage needles. First-strand cDNA was synthesized using ReverTra Ace qPCR RT Master Mix with gDNA remover according to the manufacturer’s protocol (TOYOBO, Osaka, Japan). RT-PCR was performed for *MBNL1, SORBS1* and *NFIX* with initial denaturation at 94 °C for 10 minutes and 35 cycles of 94 °C for 30 seconds, 60 °C for 30 seconds and 72 °C for 1 minutes using a thermal cycler (Applied Biosystems, California, USA), as described previously^20^,^21^. PCR products were resolved on agarose gels, stained with GelRed (Biotium, California, USA) and scanned on ChemiDoc (BIO RAD, California, USA). The density of each band was quantified using the software of ChemiDoc, Image Lab 3.0.1 (Beta 2). RT-PCR for Oct3/4, Nanog, Sox2 and β-actin were performed using the following conditions: 95 °C for 3 minutes and 30 cycles of 95 °C for 15 seconds, 58 °C for 30 seconds and 72 °C for 30 seconds, as described previously^30^. The primers used for the in-splicing analyses are listed below as follows: *MBNL1* forward: 5′-GCTGCCCAATACCAGGTCAAC-3′, *MBNL1* reverse: 5′-TGGTGGGAGAAATGCTGTATGC-3′; *SORBS1* forward: 5′-CCAGCTGATTACTTGGAATCCACGGAAG-3′, *SORBS1* reverse: 5′-GTTCTCCTTCATACCAGTTCTGATCAAT-3′; and *NFIX* forward: 5′-GAGCCCTGTTGATGACGTGTTCTA-3′, *NFIX* reverse: 5′-CTGCACAAACTCCTTCAGTGAGTC-3′. The control myocyte RNA was derived from doxycycline-inducible MyoD-healthy iPSCs (F4-MyoD, F6-MyoD and Ctr3-MyoD), as described previously^10^,^11^.

### Cell division assay

MyoD-DM1-iPSCs on the SNL feeders were used for generational tracing. A later protocol to differentiate the myocytes does neither require the feeder-free culture nor the selection of a few high mCherry-expressed clones for efficient differentiation. MyoD-DM1-iPSCs were incubated for 10 minutes with Accutase (SIGMA-ALDRICH) at 37 °C after the SNL feeders were removed using Dissociation Solution for human ES/iPS Cells (Repro CELL), dissociated into single cells and rinsed with Primate ES Cell medium (Repro CELL) supplemented with 10 μM Y-27632 (Nacalai Tesque). After the cell count and making the cell pellet, we followed the protocol of CellTrace™ Violet Cell Proliferation Kit, for flow cytometry (Thermo Fisher Scientific). The stained cells were suspended in Primate ES Cell medium (Repro CELL) supplemented with 10 μM Y-27632 (Nacalai Tesque) as well as 50 U/l penicillin/50 μg/l streptomycin (Nacalai Tesque) and seeded both at 3.0 × 10^5^ cells per 60 mm dishes for undifferentiated cell culture, which were supplemented with 8 ng/ml bFGF (Wako) as well as 100 μg/ml neomysin (Nacalai Tesque), and at 3.0 × 10^5^ (±1.0 × 10^5^) cells per well of the 24-well plate (AGC TECHNO GLASS) for myogenic differentiation, which was coated with Matrigel (Becton, Dickinson and Company) diluted 1:100 volume with Primate ES Cell medium (Repro CELL). The undifferentiated iPSCs were cultured for seven days as described above in the methods part. Myogenic differentiation of Pt-1A-MyoD or Pt-2B-MyoD required to have the medium changed to Primate ES Cell medium (Repro CELL) supplemented with 1 μg/ml or 500 ng/ml Dox (LKT Laboratories) respectively in 18 hours after the transfer. From the beginning of day 2, the myogenic differentiation was conducted as described previously^10^. On day 7, the myocytes were incubated for 5 minutes with Accutase (SIGMA-ALDRICH) at 37 °C after they were rinsed twice with PBS, rinsed with αMEM, suspended in PBS and analysed using BD LSRFortessa™ Cell Analyzer (Becton, Dickinson and Company, New Jersey, USA).

### ATAC-seq

ATAC-seq was performed as described previously^19^ with slight modifications. Living CMs were dissociated into single cells after incubating for one to two hours with collagenase I and for 20 minutes with Accutase (SIGMA-ALDRICH) at 37 °C before suspension in lysis buffer. These cells were lysed for 10 minutes in 5 to 10 μl ice-cold ATAC lysis buffer, which is 10 mM Tris-HCl pH 7.5, 10 mM NaCl, 3 mM MgCl_2_, 0.1% IGEPAL CA-630 (SIGMA-ALDRICH). Without a nuclear extraction step, Illumina’s adaptors were ligated using a transposase reaction mix of the Nextera DNA sample preparation kit (Illumina, California, USA), with incubation for 30 minutes at 37 °C. Indices were incorporated using the Nextera Index Kit (Illumina) in an amplification step. ATAC-seq libraries were purified using Ampure XP beads (Beckman Coulter Genomics, California, USA) to remove the remaining adapters. Libraries were assessed for quality and quantity using Agilent 2100 Bioanalyzer (Agilent Technologies, California, USA) and the KAPA library quantification kit for Illumina (KAPA Biosystems, Massachusetts, USA). The libraries were paired-end sequenced on the Illumina HiSeq 2500 (Illumina) for 63 cycles. Mapping was performed by BWA (ver 0.7.12), allowing up to 2 mismatches, using the reference human genome, NCBI build 37 (hg19). Then we compared the peak regions of the control CMs (648A1) and the DM1-CMs (Pt-1A) using two kinds of software, MACS2 (ver 2.1.0)^34^ and MAnorm^35^, which is the software to quantitatively compare the epigenome sequencing data sets. The parameters for MACS2 were “—nomodel —nolambda -B —extsize 100”, and its q-value cut-off was 0.05. The parameter for MAnorm was readshift_length = 0. We also compared the ATAC-seq results with the published results of CMs in the ENCODE Project^36^,^37^. In the human genome 19 (hg19), CTCF binding sites are on chromosome 19 (ch19):46273584-46273607 (CTCF_1) and ch19:46273339-46273360 (CTCF_2)^38^. Finally, we performed a quantitative assessment using MAnorm. All the above results are shown in Fig. 4 using the GenomeJack Browser (Mitsubishi Space Software Corporation, Tokyo, Japan).

## Additional Information

**How to cite this article**: Ueki, J. *et al*. Myotonic dystrophy type 1 patient-derived iPSCs for the investigation of CTG repeat instability. *Sci. Rep.* **7**, 42522; doi: 10.1038/srep42522 (2017).

**Publisher's note:** Springer Nature remains neutral with regard to jurisdictional claims in published maps and institutional affiliations.

## Supplementary Material

Supplementary Information

Supplementary Video 1

## Figures and Tables

**Table 1:** DM1-iPSCs clones and their range of repeat lengths at the establishment of the clone.

	Source	Range of repeat lengths of the source	Clone name	Range of repeat lengths at the establishment of the clone	Clone name	Range of repeat lengths at the establishment of the clone
Patient 1	Peripheral blood	200–1950	Pt-1A	300–1550	Pt-1A-MyoD	350–1600
			Pt-1B	150–1450	Pt-1B-MyoD	100–1650
Patient 2	Dermal fibroblasts	120–2050	Pt-2A	350–3100	Pt-2A-MyoD	250–3000
			Pt-2B	400–2800	Pt-2B-MyoD	200–2750
Patient 3	Dermal fibroblasts	540–3250	Pt-3A	150–2950	Pt-3A-MyoD	450–3050
			Pt-3B	150–3000	Pt-3B-MyoD	300–2900

**Table 2:** Repeat lengths of Pt-1B.

Pt-1B		Undifferentiated	CMs	Neurons
Early (P10)	Mean	998	1019	1011
	Median	1072	1127	1081
	SD	270	342	360
	CV	0.27	0.34	0.36
Middle (P21)	Mean	1169	1291	1283
	Median	1251	1408	1388
	SD	294	352	275
	CV	0.25	0.27	0.21
Late (P31)	Mean	1371	1270	1280
	Median	1513	1426	1522
	SD	411	421	431
	CV	0.30	0.33	0.34

P stands for the passage number.

**Table 3:** Repeat lengths of Pt-1B-MyoD.

Pt-1B-MyoD		Undifferentiated	Myocytes
Early (P24)	Mean	993	1036
	Median	1134	1143
	SD	328	306
	CV	0.33	0.30
Middle (P34)	Mean	1095	1071
	Median	1180	1198
	SD	294	288
	CV	0.27	0.27
Late (P47)	Mean	1262	1343
	Median	1332	1349
	SD	481	310
	CV	0.38	0.23

P stands for the passage number.
