# Supplementary material for: Myotonic dystrophy type 1 patient-derived iPSCs for the investigation of CTG repeat instability
Source: Sci Rep. 2017 Feb 13;7:42522. doi: 10.1038/srep42522 (PMC5304155; doi:10.1038/srep42522)
Supplement: Supplementary Information [file srep42522-s1.pdf]

Supplementary Information

Title: Myotonic dystrophy type 1 patient-derived iPSCs for the investigation of CTG repeat instability

Authors: Junko Ueki, Masayuki Nakamori, Masahiro Nakamura, Misato Nishikawa, Yoshinori Yoshida, Azusa Tanaka, Asuka Morizane, Masayoshi Kamon, Toshiyuki Araki, Masanori P. Takahashi, Akira Watanabe, Nobuya Inagaki and Hidetoshi Sakurai

Supplementary Fig. S1

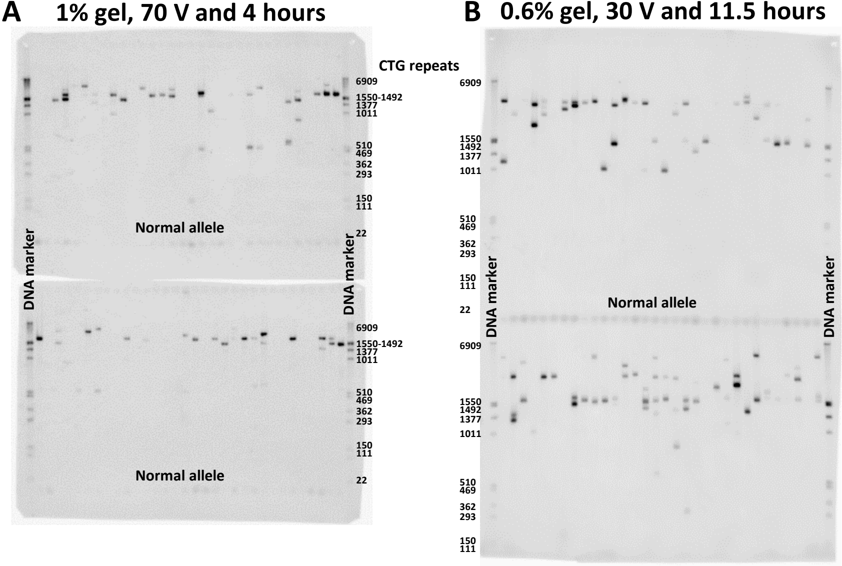

Supplementary Fig. S2

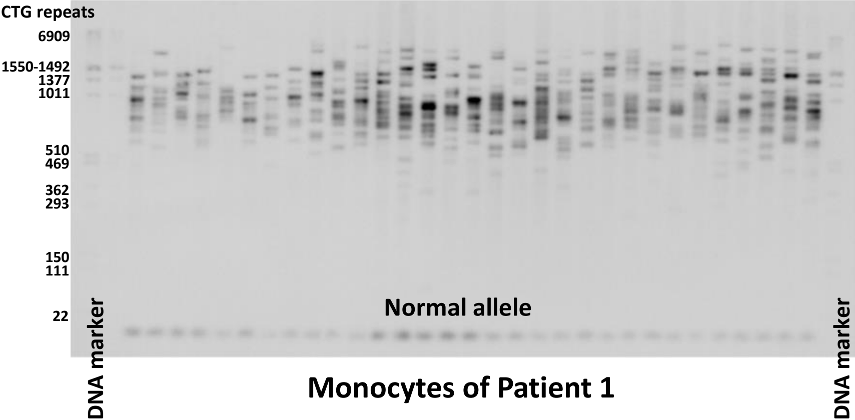

Monocytes of Patient 1

Supplementary Fig. S3   Pt-1A

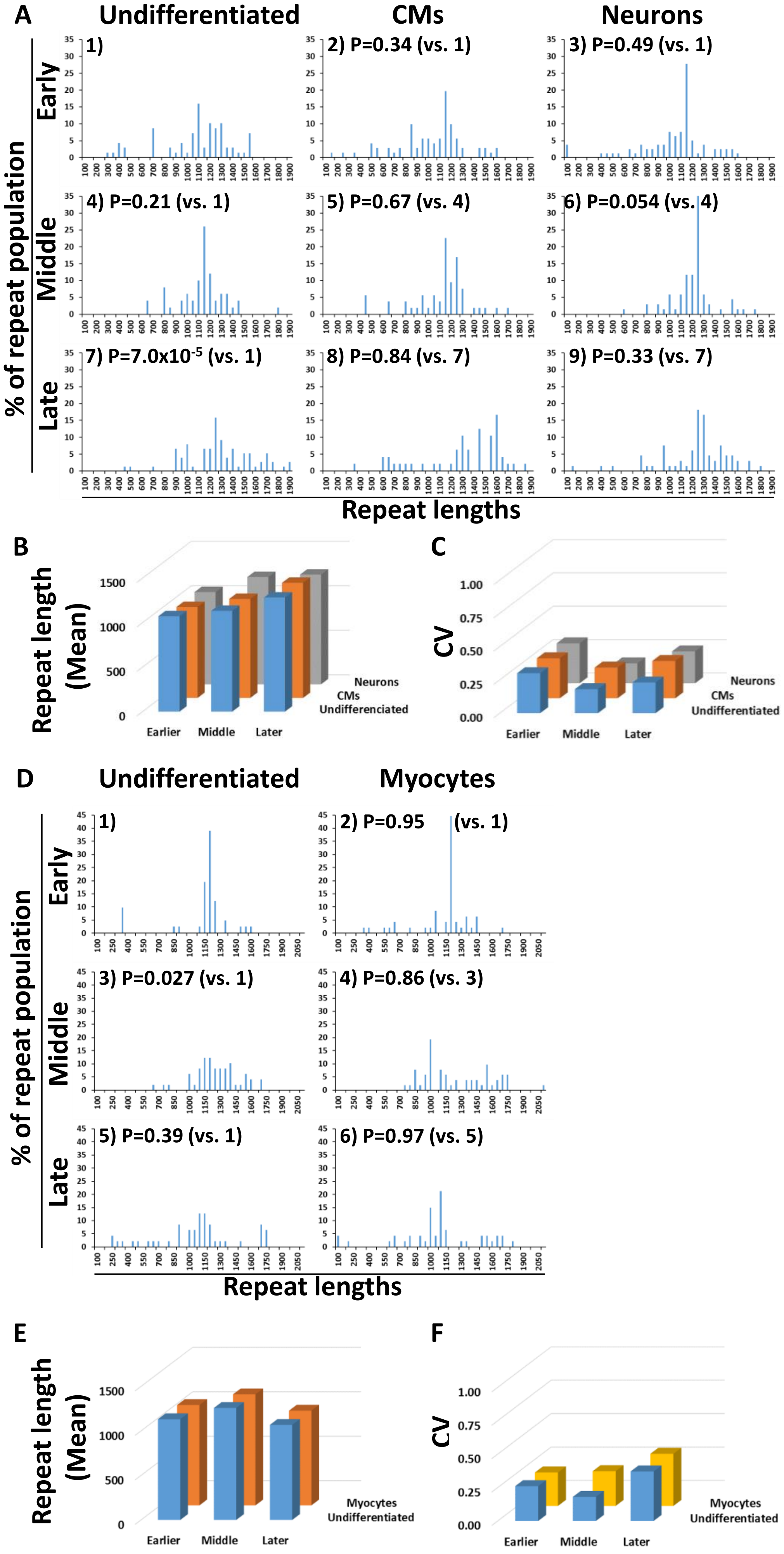

Supplementary Fig. S4 Pt-2B

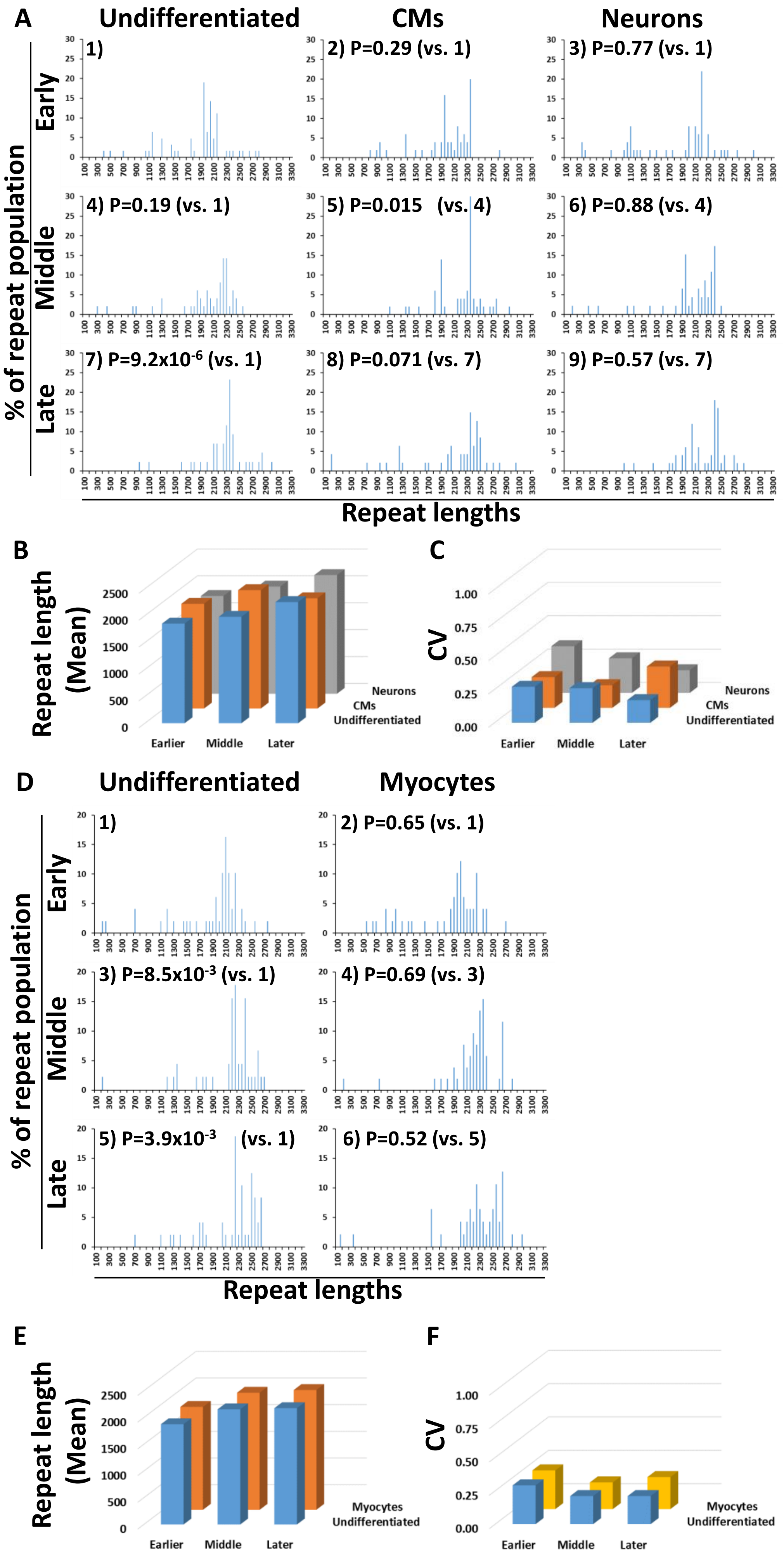

Supplementary Fig. S5 Pt-3B

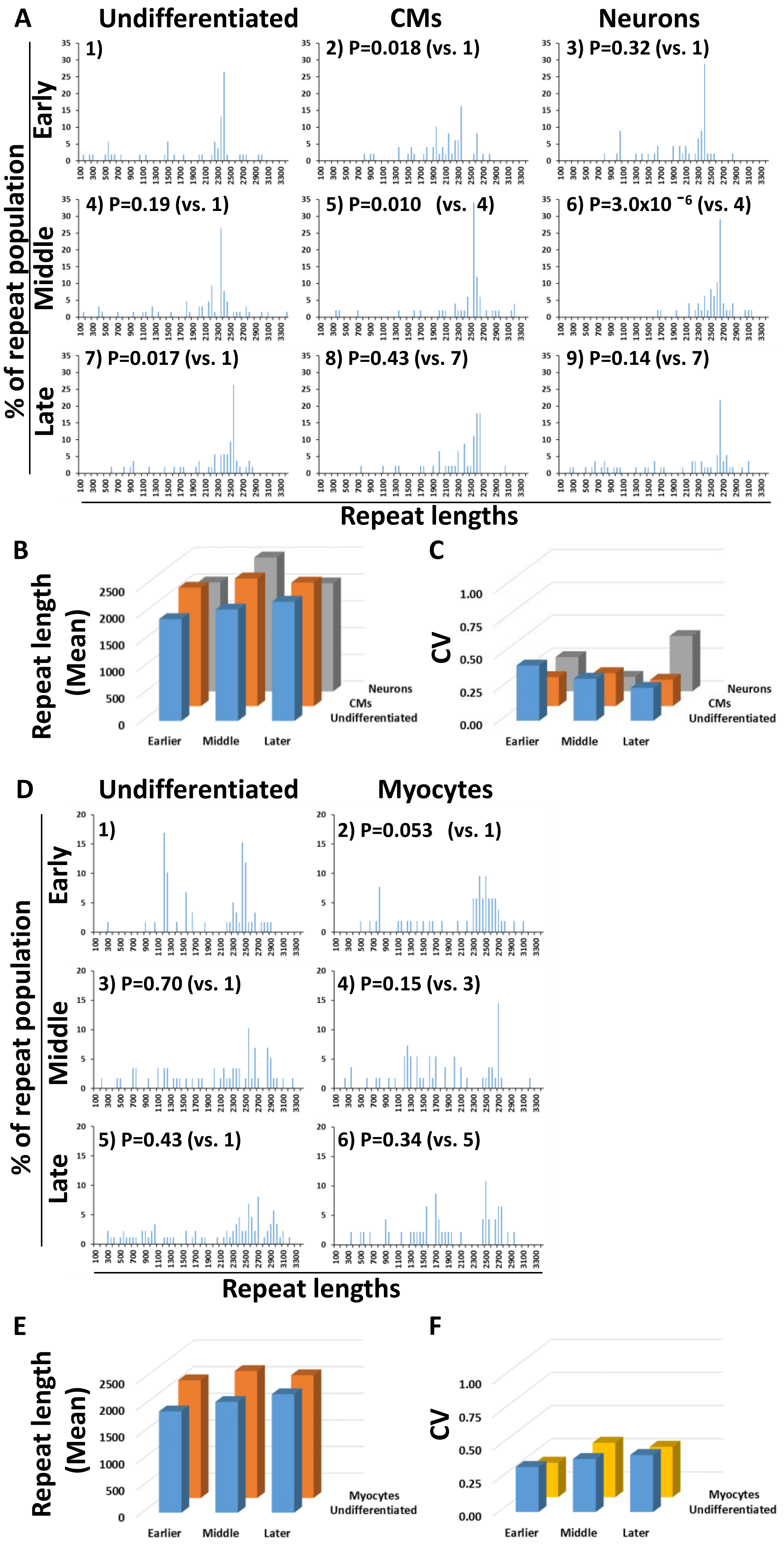

Supplementary Fig. S6

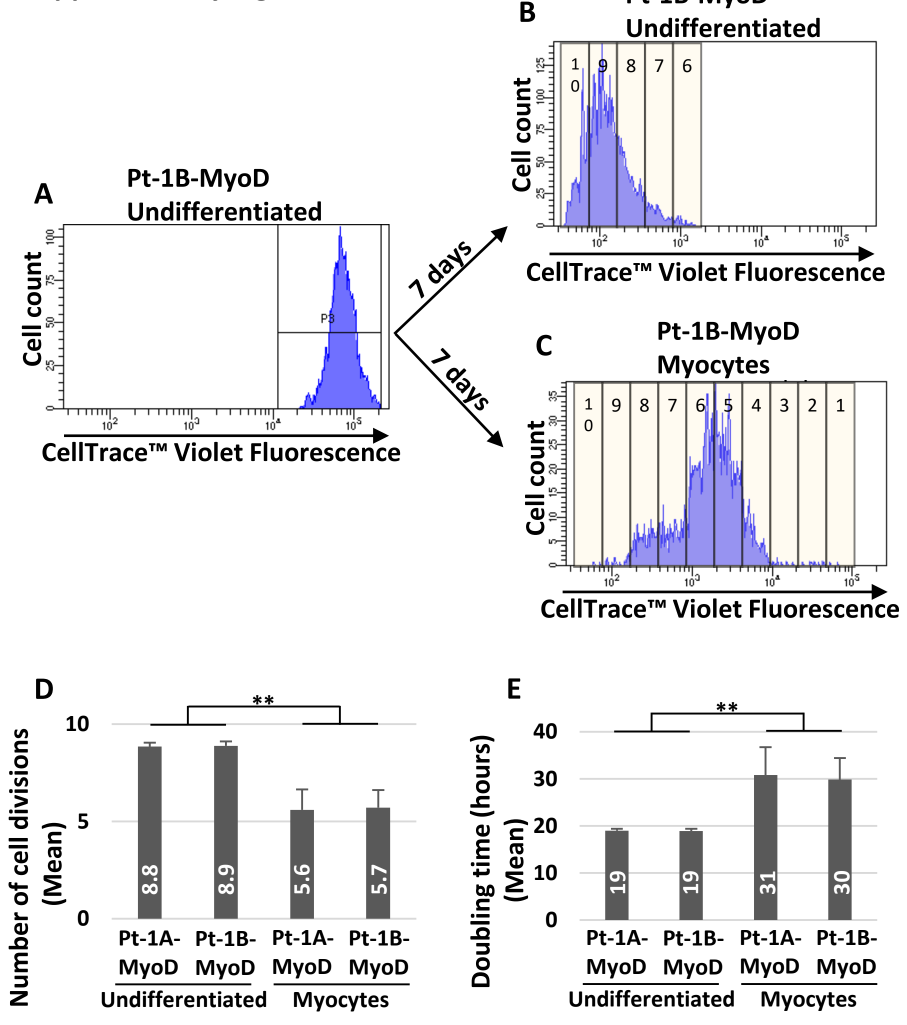

Pt-1A		Undiffer-entiated	CMs	Neurons
Early (P15)	Mean	1073	1022	1036
	Median	1117	1101	1142
	SD	318	306	311
	CV	0.30	0.30	0.30
Middle (P24)	Mean	1133	1113	1205
	Median	1149	1146	1225
	SD	207	257	186
	CV	0.18	0.23	0.15
Late (P35)	Mean	1282	1294	1233
	Median	1255	1389	1270
	SD	294	358	302
	CV	0.23	0.28	0.24
Pt-2B		Undiffer-entiated	CMs	Neurons
Early (P17)	Mean	1851	1947	1818
	Median	1966	2015	2086
	SD	496	451	644
	CV	0.27	0.23	0.35
Middle (P27)	Mean	1978	2200	1993
	Median	2190	2336	2151
	SD	508	370	527
	CV	0.26	0.17	0.26
Late (P37)	Mean	2252	2050	2207
	Median	2335	2325	2355
	SD	385	639	372
	CV	0.17	0.31	0.17
Pt-3B		Undiffer-entiated	CMs	Neurons
Early (P11)	Mean	1900	2212	2034
	Median	2354	2392	2315
	SD	794	482	519
	CV	0.42	0.22	0.26
Middle (P21)	Mean	2078	2385	2540
	Median	2328	2561	2590
	SD	661	592	290
	CV	0.32	0.25	0.11
Late (P31)	Mean	2224	2306	2017
	Median	2475	2490	2418
	SD	564	465	843
	CV	0.25	0.20	0.42

Supplementary Table S1

Pt-1A-MyoD		Undiffer-entiated	Myocytes
Early (P22)	Mean	1132	1127
	Median	1203	1199
	SD	294	279
	CV	0.26	0.25
Middle (P32)	Mean	1258	1248
	Median	1268	1137
	SD	224	327
	CV	0.18	0.26
Late (P44)	Mean	1068	1064
	Median	1086	1079
	SD	397	413
	CV	0.37	0.39
Pt-2B-MyoD		Undiffer-entiated	Myocytes
Early (P21)	Mean	1874	1924
	Median	2091	1992
	SD	545	557
	CV	0.29	0.29
Middle (P31)	Mean	2153	2190
	Median	2254	2271
	SD	461	430
	CV	0.21	0.20
Late (P42)	Mean	2175	2240
	Median	2294	2318
	SD	450	528
	CV	0.21	0.24
Pt-3B-MyoD		Undiffer-entiated	Myocytes
Early (P20)	Mean	1936	2164
	Median	2301	2448
	SD	653	569
	CV	0.34	0.26
Middle (P29)	Mean	1989	1778
	Median	2269	1675
	SD	801	733
	CV	0.40	0.41
Late (P39)	Mean	2036	1900
	Median	2387	1806
	SD	867	723
	CV	0.43	0.38

Supplementary Table S2

Figure legends

Supplementary Fig. S1 Representative image taken with LAS 4000. DNA markers were loaded into the lanes on the far left and far right of the blots. In the other 32 lanes at the top and the other 32 lanes at the bottom of the gel, spPCR products were loaded at the same time. The bands at the very bottom of the gels show a normal allele with around 20 to 35 CTG repeats. The electrophoresis conditions are shown in the figure. **(A):** Representative picture of Pt-1. The upper bands are of Pt-1B undifferentiated iPSCs, passage number 31, and the lower bands are of cardiomyocytes, differentiated from the iPSCs. **(B):** Representative picture of Pt-2 and Pt-3. The upper bands are of Pt-2A-MyoD undifferentiated iPSCs, passage number 35, and the lower bands are of myocytes on day 8, differentiated from the MyoD-iPSCs.

Supplementary Fig. S2 Monocytes from patient 1 show different lengths of CTG repeats. PCR was conducted using the same primers used for spPCR, but with higher amounts of genome. The far left and far right lanes were loaded with DNA markers.

Supplementary Fig. S3 CTG repeats of Pt-1A. **(A):** The distribution of the CTG repeats in the undifferentiated iPSCs and the CMs and neurons differentiated from the undifferentiated iPSCs at early (passages 10-17), middle (passages 21-27) and late passage numbers (passages 31-37), following the strategy shown in Fig. 1A, left. The lengths of the CTG repeats were grouped in bins spanning 50 repeats. Student's *t*-test was applied to each group of different CTG repeat lengths before being grouped in bins. *P*-values are shown. Because of the multiple comparison, the appropriate significance level was determined by Bonferroni correction, requiring a $P \leq 0.0056$ to be significant at the 95% level. P stands for *P*-values. **(B):** Mean repeat length of the nine samples. The original lengths before being grouped in bins were used to calculate the mean. The nine bar graphs correspond to 1) to 9) in Fig. S3A. **(C):** Coefficient of variation (CV) of the repeat lengths of the nine samples. CV is defined as the ratio of the standard deviation (SD) to the mean. The original lengths before being grouped in bins were used to calculate the SD. The nine bar graphs correspond to 1) to 9) in Fig. S3A. **(D):** The distribution of the CTG repeats in undifferentiated MyoD-iPSCs and in myocytes differentiated from the undifferentiated MyoD-iPSCs at early (passages 20-26), middle (passages 29-36) and late passage numbers (passages 40-48), following the strategy shown in Fig. 1A, right. The lengths of the CTG repeats were grouped in bins spanning 50 repeats. Student's *t*-test was applied to each group of different CTG repeat lengths before being grouped in bins. *P*-values are shown. Because of the multiple comparison, the appropriate significance level was determined by Bonferroni correction, requiring a $P \leq 0.0083$ to be significant at the 95% level. P stands for *P*-values. (**E), (F):** Mean and CV repeat lengths of six samples. The six bar graphs correspond to 1) to 6) in Fig. S3D.

Supplementary Fig. S4 CTG repeats of Pt-2B. **(A):** The distribution of the CTG repeats in the undifferentiated iPSCs and the CMs and neurons differentiated from the undifferentiated iPSCs at early (passages 10-17), middle (passages 21-27) and late passage numbers (passages 31-37), following the strategy shown in Fig. 1A, left. The lengths of the CTG repeats were grouped in

bins spanning 50 repeats. Student’s *t*-test was applied to each group of different CTG repeat lengths before being grouped in bins. *P*-values are shown. Because of the multiple comparison, the appropriate significance level was determined by Bonferroni correction, requiring a P ≤ 0.0056 to be significant at the 95% level. P stands for *P*-values. **(B):** Mean repeat length of the nine samples. The original lengths before being grouped in bins were used to calculate the mean. The nine bar graphs correspond to 1) to 9) in Fig. S4A. **(C):** Coefficient of variation (CV) of the repeat lengths of the nine samples. CV is defined as the ratio of the standard deviation (SD) to the mean. The original lengths before being grouped in bins were used to calculate the SD. The nine bar graphs correspond to 1) to 9) in Fig. S4A. **(D):** The distribution of the CTG repeats in undifferentiated MyoD-iPSCs and in myocytes differentiated from the undifferentiated MyoD-iPSCs at early (passages 20-26), middle (passages 29-36) and late passage numbers (passages 40-48), following the strategy shown in Fig. 1A, right. The lengths of the CTG repeats were grouped in bins spanning 50 repeats. Student’s *t*-test was applied to each group of different CTG repeat lengths before being grouped in bins. *P*-values are shown. Because of the multiple comparison, the appropriate significance level was determined by Bonferroni correction, requiring a P ≤ 0.0083 to be significant at the 95% level. P stands for *P*-values. (**E), (F):** Mean and CV repeat lengths of six samples. The six bar graphs correspond to 1) to 6) in Fig. S4D.

Supplementary Fig. S5 CTG repeats of Pt-3B. **(A):** The distribution of the CTG repeats in the undifferentiated iPSCs and the CMs and neurons differentiated from the undifferentiated iPSCs at early (passages 10-17), middle (passages 21-27) and late passage numbers (passages 31-37), following the strategy shown in Fig. 1A, left. The lengths of the CTG repeats were grouped in bins spanning 50 repeats. Student’s *t*-test was applied to each group of different CTG repeat lengths before being grouped in bins. *P*-values are shown. Because of the multiple comparison, the appropriate significance level was determined by Bonferroni correction, requiring a P ≤ 0.0056 to be significant at the 95% level. P stands for *P*-values. **(B):** Mean repeat length of the nine samples. The original lengths before being grouped in bins were used to calculate the mean. The nine bar graphs correspond to 1) to 9) in Fig. S5A. **(C):** Coefficient of variation (CV) of the repeat lengths of the nine samples. CV is defined as the ratio of the standard deviation (SD) to the mean. The original lengths before being grouped in bins were used to calculate the SD. The nine bar graphs correspond to 1) to 9) in Fig. S5A. **(D):** The distribution of the CTG repeats in undifferentiated MyoD-iPSCs and in myocytes differentiated from the undifferentiated MyoD-iPSCs at early (passages 20-26), middle (passages 29-36) and late passage numbers (passages 40-48), following the strategy shown in Fig. 1A, right. The lengths of the CTG repeats were grouped in bins spanning 50 repeats. Student’s *t*-test was applied to each group of different CTG repeat lengths before being grouped in bins. *P*-values are shown. Because of the multiple comparison, the appropriate significance level was determined by Bonferroni correction, requiring a P ≤ 0.0083 to be significant at the 95% level. P stands for *P*-values. (**E), (F):** Mean and CV repeat lengths of six samples. The six bar graphs correspond to 1) to 6) in Fig. S5D.

Supplementary Fig. S6 CellTrace™ Violet proliferation analysis. **(A):** Flow cytometric analysis of Pt-1B-MyoD undifferentiated iPSCs (Day 0) just after being stained with CellTrace™ Violet. **(B):** Flow cytometric analysis of Pt-1B-MyoD undifferentiated iPSCs after culturing for seven days. The numbers at the top indicate the number of cell divisions. **(C):** Flow cytometric analysis of myocytes seven days after differentiation from Day 0 of the undifferentiated iPSCs. The numbers at the top indicate the number of cell divisions. **(D):** The number of cell divisions of Pt-1A-MyoD and Pt-1B-MyoD after seven days in the undifferentiated or in the myogenic differentiation culture. Mean values are indicated in bars. Error bars indicate SD ($n=3$). Two-way analysis of Variance (ANOVA) was used to test differences between the undifferentiated cells group and the myocytes group based on Scheffe's test (**$p<0.01$). **(E):** The doubling time of Pt-1A-MyoD and Pt-1B-MyoD in the undifferentiated or in the myogenic differentiation culture. Mean values are indicated by bars. Error bars indicate SD ($n=3$). Two-way analysis of Variance (ANOVA) was used to test the differences between the undifferentiated cells group and the myocytes group based on Scheffe's test (**$p<0.01$).

Supplementary Table S1 Repeat lengths of Pt-1A, Pt-2B and Pt-3B. P stands for the passage number.

Supplementary Table S2 Repeat lengths of Pt-1A-MyoD, Pt-2B-MyoD and Pt-3B-MyoD. P stands for the passage number.

Supplementary Video 1 Representative video of CMs on day 20 (Pt-1B).
